# Microdialysis assessment of peripheral metabolism in critical limb ischemia after endovascular revascularization

**DOI:** 10.1186/1750-1164-3-17

**Published:** 2009-12-31

**Authors:** Matteo Tozzi, Elisa Muscianisi, Gabriele Piffaretti, Patrizio Castelli

**Affiliations:** 1 Vascular Surgery - Department of Surgical Sciences, University of Insubria, Varese University Hospital, Varese, Italy

## Abstract

**Background:**

Critical limb ischemia is a chronic pathologic condition defined by the lack of blood flow in peripheral circulation. Microdialysis is a well-known and sensitive method for the early detection of tissue ischemia. The aim of the present study was to use microdialysis in order to analyse cellular metabolism changes after peripheral endovascular revascularization.

**Methods:**

Ten patients diagnosed with critical limb ischemia was enrolled. CMA 60 (CMA^® ^- Solna, Sweden) catheter with a 20 kDa cut-off was placed subcutaneously on the anterior aspect of the foot of both limbs. Samples were collected starting 12-hours before surgery and throughout the following 72-hours, using a CMA 600 (CMA^® ^- Solna, Sweden) microdialysis analyser.

**Results:**

Technical revascularization was successful in all cases. The cannulation was well tolerated in all patients. The site of catheter insertion healed easily in few days without infective complications in any case. Two patients underwent major amputation. After revascularization, glucose showed a strong increase (mean, 5.86 ± 1.52 mMol/L, *p *= .008). No restoration of the circadian rhythm was noted in patients who underwent major amputation. Glycerol concentration curves were not deductibles in both the ischemic and the control limbs (mean, 148.43 ± 42.13 mMol/L *vs *178.44 ± 75.93 mMol/L, *p *= .348). Within the first 24-hours after revascularization, lactate concentration raised strongly (6.58 ± 1.56 mMol/L, *p *= .002): thereafter, it immediately decreased to a concentration similar to the control level (1.71 ± 1.69 mMol/L). In both patients who underwent major amputation, lactate did not show the typical peak of the successful revascularization. The trend of the lactate/pyruvate ratio after a brief initial decrease of the ratio increased again in both the patients who finally underwent amputation.

**Conclusions:**

Restoration of glucose and glycerol circadian rhythm, coupled with low lactate concentration and lactate/pyruvate ratio seemed to be linked to good surgical outcome.

## Background

Critical limb ischemia (CLI) has been recently re-defined by the Trans-Atlantic Inter-Society Consensus (TASC II) document [1,2]. Contemporarily, a vivid interest has grown on the attempt to identify biologic markers to be coupled to the usual criteria (clinical, hemodynamic, radiologic) used for diagnosis, treatment and follow-up evaluation of CLI [3].

Microdialysis is a well-known and sensitive method for early detection of tissue ischemia [4,5]. Previous studies have been published concerning microdialitic assessment of CLI in physiologic or stress-induced settings [6-8].

The aim of the present study was to investigate the modifications of some metabolites, representative of the local metabolism, during the perioperative period of peripheral revascularization in patients affected by CLI, in order to assess the potential relation between their trend and surgical outcome.

## Methods

Over a 4-months period, 10 patients (8 males) have been enrolled for this study, all diagnosed with CLI affecting just one limb. Exclusion criteria were: co-existing systemic inflammatory diseases, and previous history of revascularization in the ischemic limb. Mean age was 71 (range, 59-84). Co-morbidities and risk factors included tobacco use (n = 9), hypertension (n = 8), dislypidemia (n = 6), chronic obstructive pulmonary disease (n = 4), ischemic heart disease (n = 2), atrial fibrillation (n = 1). The grade of limb ischemia was classified accordingly to Rutherford [1], and included grade II - category 4 (n = 4) and grade III - category 5 (n = 6).

All patients underwent lower limb revascularization using endovascular percutaneous transluminal angioplasty (PTA) and/or stenting, under local anesthesia; targeted vessels were external iliac artery (n = 2) and superficial femoral artery (n = 8). During the postoperative course, the patency of the treated vessels, and run-off arteries were confirmed using Doppler-ultrasonography. All patients received hydric diet during preoperative period; low molecular weight heparin was administered during the postoperative hospitalization.

The catheter (CMA 60-CMA^®^-Solna, Sweden) of the microdialysis consisted of a dialyzing polyamide membrane 30 mm in length, 0.5 mm in diameter, and molecular weight cut-off of 20 kDa. Perfusion was guaranteed by a variable flux pump CMA 106 (CMA^®^-Solna, Sweden) that administered isotonic saline solution (Na^+ ^147 mMol, K^+ ^4 mMol, Ca^2+ ^2.3 mMol, Cl^- ^156 mMol, 290 mosm/kg) into the probe inserted in the targeted tissue with stable velocity for the entire procedure. Perfusion fluid flow was maintained at 0.3 μL/min. Ultra-filtrated dialyzed solution has been examined using an enzymatic and colorimetric method for glucose, glycerol, lactate and pyruvate (CMA 600 Microdialysis Analyser-CMA^®^-Solna, Sweden). Evaluated parameters were intended from the interstitial fluid, and not from peripheral blood.

The catheter was allocated in the subcutaneous tissue into the anterior portion of the foot below the ankle. No stress tests have been performed, and both limbs lied horizontal for the entire procedure. Local anesthesia (Carbocaine^® ^1%-AstraZeneca-Caponago, Milano-Italy), was used to introduce the catheter into the tissue with a slit cannula introducer, leaving the dialyzing polyamide membrane "in situ" when the samples were obtained; this was performed about 12-hours before revascularization The vials were changed at 3-hours intervals pre- and postoperatively, and on an hourly basis throughout the intraoperative stay. "Positive control" was placed into the controlateral limb in the same position.

Referral physiologic levels was considered accordingly to what reported by Rosdahl et al. [9] (glucose 4.12 ± 0.31 mMol, of lactate 1.30 ± 0.12 mMol, glycerol 68.7 ± 3.97 μMol). Clinical data were recorded and tabulated with Microsoft Excel^® ^(Microsoft Corp, Redmond, WA, USA). Statistical analysis was computed with SPSS, release 16.0 for Windows (SPSS Inc.^®^, Chicago, IL, USA). Results were expressed as mean ± SD for continuous variables and frequencies for the categorical ones. A 2-sided *p *value less than .05 was considered statistically significant. Variables and values were compared between groups with unpaired Student's *T *test and the Wilcoxon test as appropriate.

## Results

Technical revascularization was successful in all cases. The cannulation was well tolerated in all patients. The site of catheter insertion healed easily in few days without infective complications in any case. Two patients underwent major amputation.

**Figure 1:**
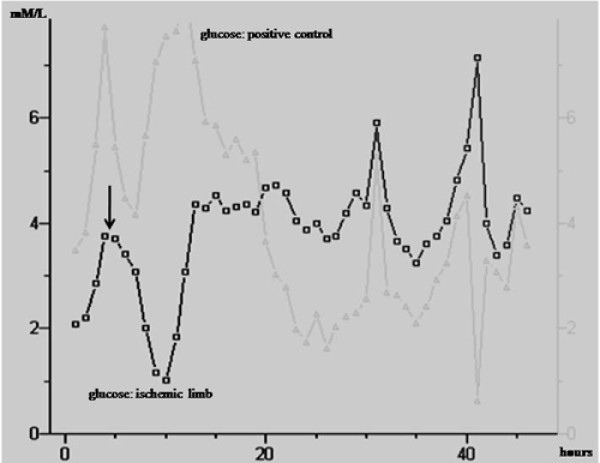
**Ischemic *vs *positive control: comparative analysis of the glucose trend in one patient**. The basal level in the ischemic limb was perceptible lower than the positive control. After revascularization (*arrow*), glucose level in the ischemic limb rapidly decreased and finally re-increased reaching similar trend of the positive control: the circadian rhythm was restored. Note that the important variations in the positive control could be influenced by the neuroendocrine stimulation.

During preoperative ischemic phase, glucose concentration lied on a lower baseline in the treated limb if compared to the controlateral (mean, 1.61 ± 1.35 mMol/L *vs *4.03 ± 0.63 mMol/L, *p *= .001). In the ischemic limb, circadian rhythm was not observed. After revascularization, glucose showed a strong increase (mean, 5.86 ± 1.52 mMol/L, *p *= .008). Circadian rhythm, in relation to diet and morning neuroendocrine stimulation, was restored with a trend and a pace almost identical to the positive control within 24-30 hours after surgery (Figure 1). No restoration of the circadian rhythm was noted in patients who underwent major amputation.

**Figure 2:**
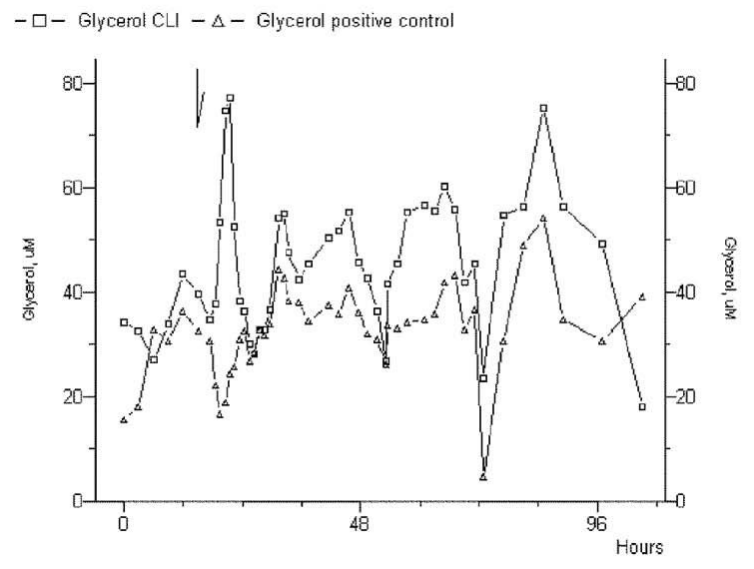
**Glycerol analysis in a patient who underwent successful revascularization**. As yet described in the text, the circadian rhythm was restored in those patients post-procedurally, and the alternated peaks were similar in the treated limb versus the control limb.

**Figure 3:**
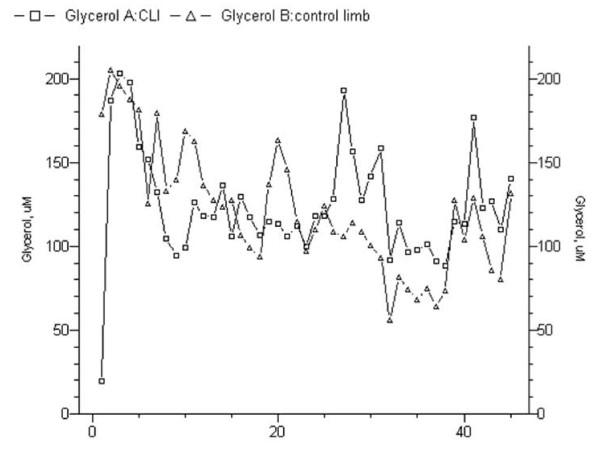
**Glycerol analysis in a patient whose revascularization failed**. In this case it was not evident the restoration of the circadian rhythm and the not coupled curves of the treated limb versus the control limb.

Glycerol concentration curves were not deductibles in both the ischemic and the control limbs (mean, 148.43 ± 42.13 mMol/L *vs *178.44 ± 75.93 mMol/L, *p *= .348). It exhibited alternated peaks, either in pre- and postoperative period (Figure 2). Standard deviation expresses a highly disordered tendency; however, after revascularization, every peak paired those of the control limb, demonstrating the restoration of circadian rhythm. Both patients who underwent limb amputation did not show similarity of peaks in both legs within 72-hours from the intervention (Figure 3).

**Figure 4:**
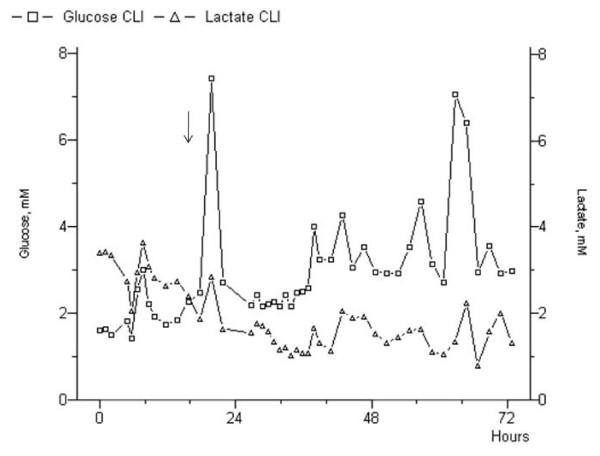
**Glucose and lactate curves in a patient who had successful revascularization**. Note the immediate peak of the lactate post-procedurally (*arrow*), with the specular reduction of the glucose possibly testifying the restoration of the cellular metabolism.

**Figure 5:**
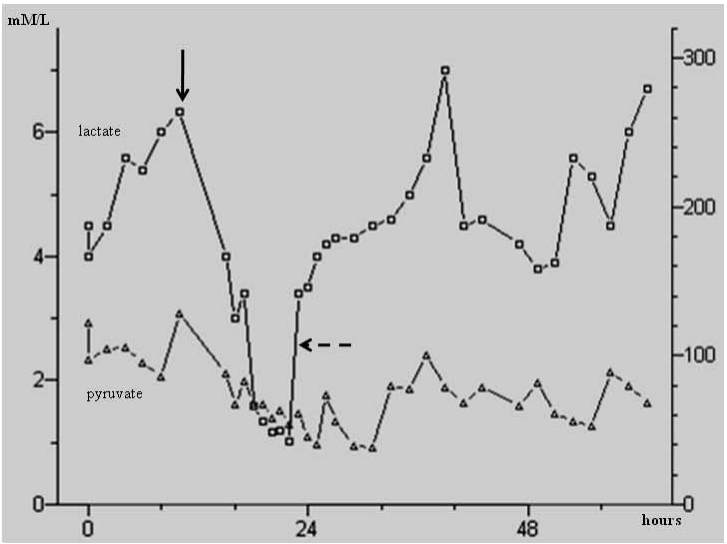
**Lactate and pyruvate curves in the ischemic limb of a single patient**. After the first attempt of revascularization (*arrow*), lactate decreased. After a short period, lactate started to rise again (*sketched arrow*): this patient experienced a recurrent thrombosis of the treated arterial district, and finally underwent major amputation.

During the preoperative ischemic phase, lactate concentration lied on a higher baseline in the ischemic limb if compared to the controlateral (mean, 3.68 ± 1.61 mMol/L *vs *1.54 ± 1.36 mMol/L, *p *= .01). Within the first 24-hours after revascularization, lactate concentration raised strongly (6.58 ± 1.56 mMol/L, *p *= .002) because of the oxygen restoration and the increased aerobic metabolism, also testified by the decreased of the glucose level (Figure 4). Thereafter, lactate immediately decreased to a concentration similar to the control level (1.71 ± 1.69 mMol/L). In both patients who underwent major amputation, lactate did not show the typical peak of the successful revascularization; in particular, a consistent decrement (< 1.74 mMol/L) was observed early after revascularization, but within 12-hours both patients presented an elevation of lactate comparable to the preoperative ischemic phase (Figure 5).

**Figure 6:**
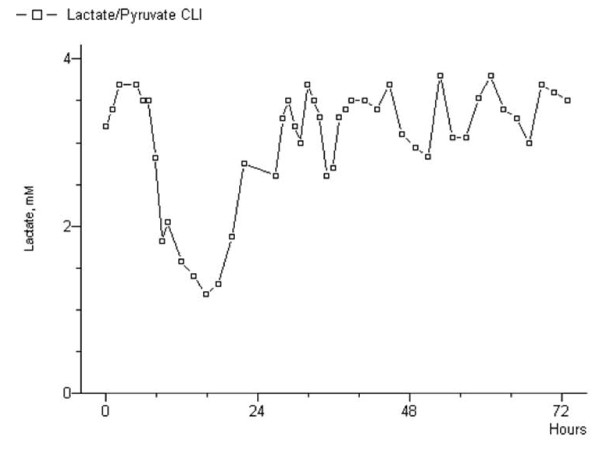
**Lactate/Pyruvate ratio in the ischemic limb of the patient presented in figure 5**. The reduced ratio confirmed the initial success of the surgical procedure. After a short period, lactate/pyruvate ratio started to rise again. Note the persisting high level of the ratio for recurrent ischemia.

The pyruvate quantification showed an almost equivalent concentration in both legs, even if slightly elevated in the ischemic limb when compared with the positive control (98.4 ± 13.3 *vs *96.5 ± 14.22 μMol/L, *p = *.786). Pyruvate evaluation was important to evaluate the lactate/pyruvate ratio: the trend of this ratio was similar to what observed for lactate, with a rapid decrease after the revascularization procedure. We noted that, after a brief initial decrease of the ratio, it increased again in both the patients who finally underwent amputation (Figure 5, 6). We have not evaluated the pyruvate only, because it suffered the influence of the neuroendocrine activity and the circadian rhythm most importantly than any other substances.

## Discussion

Critical limb ischemia is generally considered the end-stage of peripheral arterial obstructive disease, one of the most common manifestations of atherosclerosis; this is a pathological, distinctive condition because of its progressive and persistent lack of blood and oxygen [1]. The consequences of prolonged ischemia, are cellular death and tissue necrosis as a result of hypoxia and energetic substrates depletion, finally leading to limb loss [2,3,10]. Hence, it should be attractive to identify some specific markers that can be useful especially to optimize diagnosis, and to evaluate the outcomes of the revascularization procedures. The main finding of this study was a kind of "metabolic" state characterizing CLI and early postoperative course after endovascular revascularization.

First proposed in late '60s for neurological applications, microdialysis has been adapted to test different tissues [11,12]. Microdialysis is a well-established technique for the continuous sampling of small water-soluble molecules within the extracellular space in vivo. Sampling rationale is based on the passive diffusion of substances across a semi-permeable hollow-fiber dialysis membrane driven by a concentration gradient and supported by a continuous flux pump simulating the function of a vein [13]. Substances smaller than the membrane pores can diffuse into the inner lumen of the catheter and be carried to the outlet by the perfusion fluid in relation to membrane pores and velocity. Larger substances will either be completely rejected by the membrane pores or diffuse so slowly through these pores causing their recovery to be negligible. Our choice of 20 kDa pores supported by a 0.3 μL/min fluid flow was dictated by the will to obtain a recovery proximal to 100% of the third space, in order to evaluate the concentration of metabolic markers that directly reflects the effective status of the intracellular metabolism [9,14,15].

Similar analysis on CLI metabolism and its modification during revascularization was performed by Metzsch et al. [5]: the authors tested glycerol, glucose and lactate into subcutaneous and muscular tissue of ischemic limb, adopting pectoral subcutaneous tissue as "positive control". Our study aimed to define specifically the clinical condition of these metabolites in CLI, that's why we opted for the non ischemic controlateral limb as control. Indeed, we estimate the subcutaneous tissue of the most distal part of the leg because this peripheral segment is thought to be the most affected, and sensitive to the ischemic condition; in fact, in CLI blood flow is extremely reduced in this area, therefore the metabolic agent derived from the samples are really emblematic of a pathologic condition. In addition, our study significantly differed since we used two identical location than three different sites, we provided a higher number of samples in a more extensive period of time, and most importantly we had amputations that meant a subgroup of control for the different substances.

The lactate in our series presented similar absolute levels and curves to what published in the experience of Metzcsh et al. [5], but glucose and glycerol demonstrated different patterns. In their experience, glucose was influenced by the neuroendocrine stress response that induced a preoperative growth of the baseline level; in contrast to their findings, our glucose levels rapidly decreased in the post-procedural period as a possible consequence of the restoration of an aerobic metabolism. The glycerol in our group showed a lower baseline level. However, it should be taken into account that our cases were affected by critical (and not chronic) limb ischemia.

In their study, Rosdahl et al. [9] tested the same substances in physiologic condition, and demonstrated that glucose concentration was equivalent in plasma and in interstitium, whereas plasmatic concentrations of lactate and glycerol were lower than interstitial concentration found in adipose and muscle tissues. In our study we evaluated a chronic disease; we registered an ischemic preoperative condition distinguished by a lower glucose concentration coupled by a concentration higher lactate level than those observed in physiologic condition. Also, in postoperative period we found a tendency of glucose to rise, whereas lactate decreased after surgery with values tending to adapt to physiologic normality. This was supposed to be related to a slow rehabilitation to the newly restored aerobic metabolism. Glucose and glycerol re-establishment of circadian rhythm was detected in all cases with good early outcome. However, we should advise that the influence of adrenergic stimulation cannot be excluded as confounding factor.

Of interest, both patients who experienced limb loss did not present a decrement of lactate concentration, that tended to persist at high levels if compared to the pyruvate baseline (persistence of ischemia), and did not present an increase of the glucose interstitial concentration. This was hypothesized to be the result of the persistence of an anaerobic metabolism supporting the cellular need in front of the lack of oxygen.

The present study deserves a brief final specification: we posed careful attention to avoid additional trauma to the ischemic limb because the system, left in place for several hours even with minimal trauma, would potentially expose to bacterial agents [9,15]. Of note, our procedures healed easily in few days without infective complications, and the catheterization was well tolerated in all cases.

## Conclusions

The main defect of this study was the limited number of patients, along with the potential confounding variables represented by the neuroendocrine response and the short preoperative period of observation. However, in this opening experience microdialysis revealed to be a potential attractive tool to define the metabolic response distinguishing CLI (absence of circadian rhythm of glucose and glycerol, high lactate and lactate/pyruvate ratio), and also to detect quickly the potential persistence or absence of local ischemia after revascularization.

## Competing interests

The authors declare that they have no competing interests.

## Authors' contributions

MT: concept; study design; carried out the analytic procedures; data analysis; writing.

EM: data collection; writing.

GP: data analysis; writing.

PC: concept; study design; data analysis.

All authors read and approved the final manuscript.
